# Development and Characterization of Alkaline Phosphatase-Positive Human Umbilical Cord Perivascular Cells

**DOI:** 10.3390/cells10113011

**Published:** 2021-11-04

**Authors:** Shun Nonoyama, Takeo Karakida, Risako Chiba-Ohkuma, Ryuji Yamamoto, Yuko Ujiie, Takatoshi Nagano, Yasuo Yamakoshi, Kazuhiro Gomi

**Affiliations:** 1 Department of Periodontology, School of Dental Medicine, Tsurumi University, 2-1-3 Tsurumi, Tsurumi-ku, Yokohama 230-8501, Japan; 3011004@stu.tsurumi-u.ac.jp (S.N.); 1yam3785@jcom.home.ne.jp (Y.U.); nagano-takatoshi@tsurumi-u.ac.jp (T.N.); gomi-k@tsurumi-u.ac.jp (K.G.); 2 Department of Biochemistry and Molecular Biology, School of Dental Medicine, Tsurumi University, 2-1-3 Tsurumi, Tsurumi-ku, Yokohama 230-8501, Japan; karakida-t@tsurumi-u.ac.jp (T.K.); chiba-r@tsurumi-u.ac.jp (R.C.-O.); yamamoto-rj@tsurumi-u.ac.jp (R.Y.)

**Keywords:** human umbilical cord perivascular cell, alkaline phosphatase, transforming growth factor-beta, fibroblast, myofibroblast

## Abstract

Human umbilical cord perivascular cells (HUCPVCs), harvested from human umbilical cord perivascular tissue, show potential for future use as an alternative to mesenchymal stromal cells. Here, we present the results for the characterization of the properties alkaline phosphatase-positive HUCPVCs (ALP(+)-HUCPVCs). These ALP(+)-HUCPVCs were created from HUCPVCs in this study by culturing in the presence of activated vitamin D3, an inhibitor of bone morphogenetic protein signaling and transforming growth factor-beta1 (TGF-β1). The morphological characteristics, cell proliferation, gene expression, and mineralization-inducing ability of ALP(+)-HUCPVCs were investigated at the morphological, biological, and genetic levels. ALP(+)-HUCPVCs possess high ALP gene expression and activity in cells and a slow rate of cell growth. The morphology of ALP(+)-HUCPVCs is fibroblast-like, with an increase in actin filaments containing alpha-smooth muscle actin. In addition to ALP expression, the gene expression levels of type I collagen, osteopontin, elastin, fibrillin-1, and cluster of differentiation 90 are increased in ALP(+)-HUCPVCs. ALP(+)-HUCPVCs do not have the ability to induce mineralization nodules, which may be due to the restriction of phosphate uptake into matrix vesicles. Moreover, ALP(+)-HUCPVCs may produce anti-mineralization substances. We conclude that ALP(+)-HUCPVCs induced from HUCPVCs by a TGF-β1 stimulation possess myofibroblast-like properties that have little mineralization-inducing ability.

## 1. Introduction

Mesenchymal stem cells (MSCs) are a type of somatic stem cell derived from mesenchymal tissues such as bone marrow [1,2], the adipose tissue stromal vascular fraction [3,4], the placenta [5], and the umbilical cord [6,7]. MSCs possess self-replication and multilineage differentiation abilities and pose few risks for tumor formation, so they are expected to be a cell source for regenerative medicine. The MSCs isolated from each tissue have similar expression profiles and biological functions for cell surface markers but possess different characteristics depending on the tissues from which they were derived. Bone marrow MSCs have been widely used for both basic and clinic studies because of their ease of collection and high proliferative potential [2,8,9]. Greater quantities of immature MSCs can be obtained from fetal appendages such as Wharton’s jelly in the umbilical cord and placental amniotic membrane [10,11,12]. Human umbilical cord perivascular cells (HUCPVCs) harvested from human umbilical cord perivascular tissue are an MSC population and have been used as a cell source for dermal wound healing [13,14] and other MSC studies [15,16,17]. HUCPVCs area mesenchymal cell population with a high colony-forming unit-fibroblast frequency at harvest is 1:300, which is considerably higher than that of bone marrow, at 1:100,000. In addition, the collection of MSCs from bone marrow is invasive and painful, while that of HUCPVCs is non-invasive. Therefore, HUCPVCs are expected to be used as an alternative to MSCs in the future. In vitro studies using conditioned media prepared from cultures of human bone marrow derived mesenchymal stromal cells showed that HUCPVCs have the potential for osteogenic differentiation [18,19]. Alkaline phosphatase (ALP) is found widely localized in vivo in various tissues such as the small intestinal mucosa, placenta, testis, liver, kidney, bone, and fibroblast [20]. As ALP is highly expressed in undifferentiated embryonic stem cells, induced pluripotent stem cells, and hard tissue-forming cells such as osteoblasts and odontoblasts, its enzymatic activity is used as a marker of differentiation [21,22,23]. Our previous study showed that intrinsically high ALP activity in cells is required to differentiate dental pulp cells into odontoblasts [24].

Here, we investigated the characteristics of ALP-positive HUCPVCs at the biological, histochemical, immunohistochemical protein, and genetic cell levels.

## 2. Materials and Methods

This study was approved by the Ethics Committee of Tsurumi University School of Dental Medicine, (approval No. 1011) and was conducted in accordance with the Guidelines on clinical research using human stem cells, the notification of which was issued by the Ministry of Health, Labour, and Welfare. HUCPVCs were prepared by the same procedure as Kajiyama et al. (Appendix A) [19].

### 2.1. Alkaline Phosphatase (ALP) Activity Assay

The HUCPVCs were plated on a 96-well plate at a density of 1.0 × 10^4^ cells/well and were cultured with the standard medium composed of alpha minimum essential medium (αMEM; Thermo Fisher Scientific, Waltham, MA, USA) containing 10% fetal bovine serum (FBS), 50 U/mL penicillin, and 50 μg/mL streptomycin (Thermo Fisher Scientific) in a humidified 5% CO_2_ atmosphere for 24 h at 37 °C. The FBS used in this experiment was deactivated by incubation at 56 °C for 30 min and was added into the medium to a concentration of 10%. The medium was changed to a mineralization medium supplemented with various combinations of 1 ng/mL recombinant human transforming growth factor beta-1 (TGF-β1) (Cell Signaling Technology, Danvers, MA, USA); 500 ng/mL recombinant human bone morphogenic protein-2 (BMP-2) (#355-BEC, R&D Systems, Minneapolis, MN, USA); 50 nM activated vitamin D3 (VD) (EMD Millipore Corp., Billerica, MA, USA) 50, 100 or 200 nM LDN-193189 (LDN) (Tocris Bioscience, Bristol, UK); 1 μM SB431542 (SB) (ChemScene, Monmouth Junction, NJ, USA); and 100 nM dexamethasone (DEXA) (MilliporeSigma, St. Louis, MO, USA). The ALP activity in each well was measured based on our previous method [25]. The cells were washed once with phosphate-buffered saline (PBS) on days 3 and 7, and ALP activity was determined using the following ALP reaction solution: 10 mM p-nitrophenylphosphate as the substrate in a 100 mM 2-amino-2-methyl-1,3-propanediol-HCl buffer (pH 10.0) containing 5 mM MgCl_2_ and incubated for 10 min at 37 °C. We added 0.2 M NaOH to quench the reaction, and the absorbance at 405 nm was read on a plate reader. Since the cells were confluent in the well of the 96-well culture plate at the time of measurement, we assumed that the number of cells in each well was constant, and the 405 nm absorbance of the reaction solution in each well was adopted as the index for ALP activity.

### 2.2. Morphological Observation of HUCPVCs

#### 2.2.1. Alkaline Phosphatase Staining

HUCPVCs were stained with 0.1 mg/mL of naphthol AS-MX phosphate (N AS-MX P, Sigma-Aldrich, St. Louis, MO, USA), 0.5% N,N-dimethylformamide, 0.6 mg/mL of Fast blue BB salt (Sigma-Aldrich), and 2 mM MgCl_2_ in 0.1 M Tris-HCl buffer (pH 8.5) for 30 min at room temperature and then washed with dH_2_O and photographed.

#### 2.2.2. Rhodamine Phalloidin–DAPI Staining

The HUCPVCs were treated with PBS containing 0.1% Triton-X for 30 min at room temperature and rinsed with PBS at 3 times. The cells were stained with 100 nM rhodamine phalloidin and 2 μg/mL of 4′,6-diamidino-2-phenylindole (DAPI) in PBS for 5 min at room temperature and then washed with PBS. The cells were observed using a fluorescence microscope (Biozero BZ-8100, Keyence, Osaka, Japan).

#### 2.2.3. Fluorescent Immunostaining 

HUCPVCs were grown on 8-well chamber slides (Matsunami Glass IND. Ltd., Osaka, Japan) at a density of 1.0 × 10^3^ cells/well and cultured in a standard medium for 7 days, changing the medium every day. The cells were fixed with 4% paraformaldehyde for 15 min at room temperature and incubated in a blocking solution (1% BSA, 10% normal goat serum) for 1 h at room temperature. For the primary antibody application, an anti-elastin monoclonal antibody (#ab9519, Abcam, Cambridge, UK), anti-fibrillin-1 polyclonal antibody (#BS-1157R, Bioss Antibodies Inc., Woburn, MA, USA), anti-osteopontin polyclonal antibody (#AF808, R&D Systems, Minneapolis, MN, USA), and anti α-SMA antibody (#23081-1-AP, Proteintech Japan, Tokyo, Japan) were used at 1:200 or 1:300 dilutions, and the cells were incubated in the above antibody mixture overnight at 4 °C. For the secondary antibody application, Alexa Fluor^TM^ 546 rabbit anti-mouse IgG (H+L) and Alexa Fluor^TM^ 488 goat anti-rabbit IgG (H+L) antibodies (Thermo Fisher Scientific) were used at a dilution of 1:500, and the cells were incubated for 1 h at room temperature while protected from light. DAPI was used to stain the cell nuclei as the counterstaining at a concentration of 2 μg/mL. Light micrographs were obtained using a Canon EOS Kiss X8i camera (Canon, Tokyo, Japan) on an optical microscope (OLYMPUS BX50, Olympus, Tokyo, Japan). The merged image was created using ImageJ software Version 1.52a (National Institutes of Health, Bethesda, MD, USA).

### 2.3. Cell Proliferation Assay

The HUCPVCs were plated into 96-well plates at a density of 1.0 × 10^3^ cells/well for cell proliferation assay (MTS assay) in standard media supplemented with or without TGF-β1, VD, and LDN, and cultured at 37 °C in a humidified 5% CO_2_ atmosphere. The culture medium was changed every day. The proliferation rate of the cells in six 96-well plates was determined on days 1, 2, 3, 4, 5, 6, and 7 using a CellTiter 96^®^AQueous One Solution Cell Proliferation Assay (Promega Corporation, Madison, WI, USA). The cell population doubling time was calculated using a regression curve.

### 2.4. Quantitative Polymerase Chain Reaction (qPCR) Analysis

The cells were extracted with an RNA extraction kit (Roche Diagnostics GmbH, Mannheim, Germany). The total RNA (1 μg) was purified and reverse-transcribed followed by the reaction mixture consisting of SYBR green PCR master mix (Roche Diagnostics GmbH) supplemented with 0.5 µM forward and reverse primers and 2 µL of cDNA as the template. The specific primer sets were designed using Primer-BLAST (National Institutes of Health) as the primer designing tool [20]. Samples were run for 45 cycles (denaturation for 10 sec at 95 °C, annealing for 10 sec at 60 °C, and extension for 15 sec at 72 °C). The specific primer sets and running conditions are shown in Table A1. Glyceraldehyde-3-phosphate dehydrogenase (*GAPDH*) was used as the reference gene. Each ratio was normalized to the relative quantification data of alkaline phosphatase (*ALP*), collagen type I alpha 1 chain (*COL I*), osteopontin (*OPN*), osteocalcin (*OC*), runt-related transcription factor 2 (*RUNX2*), elastin (*ELN*), fibrillin-1 (*FBN-1*), myoblast determination protein 1 (*MYOD1*), myogenic factor 5 (*MYF5*), periostin (*POSTN*), periodontal ligament associated protein-1 (*PLAP1*), cluster of differentiation 73, 90, and 105 (*CD73*, *CD90* and *CD105*), α-smooth muscle actin (*α-SMA*), and phosphate transporter 1 (*PiT1*) in comparison to the *GAPDH* that was generated on the basis of a mathematical model for relative quantification in the qPCR system.

### 2.5. Detection of Mineralized Nodules in HUCPVC 

HUCPVCs were grown on a 12-well plate at an initial density of 3.16 × 10^4^ cells/cm^2^. After incubation for 24 h, the medium was changed to a mineralization-inducing medium containing 10 mM β-glycerophosphate and 50 μM ascorbic acid (mineralization medium) supplemented with or without TGF-β1, VD, and LDN. The cells were cultured for up to 21 days. Mineralization was visualized by Alizarin Red S staining. After fixation with 4% paraformaldehyde neutral buffer solution for 10 min, the cells were stained with a 1% Alizarin Red S solution (Sigma-Aldrich) for 10 min, then washed with distilled water and photographed. This experiment was repeated 3 times, and the full image of all results that were cropped in the main figure is shown in Figure A2.

### 2.6. Characterization of Matrix Vesicles of HUCPVCs

#### 2.6.1. Fractionation of Matrix Vesicles 

Matrix vesicles (MVs) were prepared by a modification to the method of Hayashi et al. [26]. HUCPVCs were grown on a 12-well plate at an initial density of 3.16 × 10^4^ cells/cm^2^. After incubation for 24 h, the medium was changed to a mineralization-inducing medium and cultured with or without VD, LDN, and TGF-β1 for 9 days. The cells were washed with 20 mM Tris-buffered saline (TBS) (pH 7.5) and digested with 0.45% collagenase for 1 h at 37 °C. The digest was centrifuged at 2500 rpm for 10 min at 4 °C and the pellet was designated whole cells or “Cells”. The supernatant was further centrifuged at 22,000 rpm for 10 min at 4 °C and the pellet was designated organelles or “Org”. The supernatant was finally centrifuged at 40,000 rpm for 2 h at 4 °C and the pellet was designated matrix vesicles or “MVs”. Three pellets, Cells, Org, and MVs were suspended with 20 mM TBS (100 μL for Cells and 50 μL for Org and MVs) and used for the experiments described below.

#### 2.6.2. ALP Activity Assay

Three pellets (5 μL each) in 96-well plates were mixed with 100 μL of ALP reaction solution described in Section 2.1. and incubated for 6 min at 37 °C. We added 0.2 M NaOH (50 μL) to quench the reaction, and the absorbance at 405 nm was then read on a plate reader.

#### 2.6.3. Measurement of Ca Content

Three pellets (5 μL each) were mixed with 2.5 μL of 0.5 N HCl in a tube and left to stand for 10 min. Samples were centrifuged at 13,000 rpm for 5 min at 4 °C and the supernatant (2.5 μL) was transferred into a 96-well plate. The calcium concentration in the supernatant was spectrophotometrically determined at 595 nm by following the color development with a calcium assay kit (Calcium E-test Wako, Fujifilm Wako Pure Chemical Industries, Ltd., Osaka, Japan). All values were normalized against the cultivation area.

#### 2.6.4. Western Blotting

SDS-PAGE was performed using 5–20% e-PAGEL mini gel (ATTO Corporation, Tokyo, Japan) electrotransferred onto PVDF membrane (Thermo Fisher Scientific). Following blocking with Blocking One solution (ATTO Corporation) for 1 h at room temperature, polyclonal anti-PiT1/SLC20A1 antibody (GTX105062, Gene Tex, Irvine, CA, USA) was used at a dilution of 1:500 and reacted overnight at 4 °C. The membrane was reacted against goat anti-rabbit IgG (BioRad, Hercules, CA, USA) at a dilution of 1:2000 for 1 h at room temperature. The membrane was immunostained by chemiluminescent detection with ECL Prime (GE Healthcare. Uppsala, Sweden). The full image of the blots that were cropped in the main figure is shown in Figure A2. The band intensity was calculated using ImageJ software Version 1.52a (National Institutes of Health, Bethesda, MD, USA).

### 2.7. Search for Calcification Inhibitor Produced by HUCPVCs

A porcine dental pulp-derived cell line (PPU7) that we previously established [24] was used for this experiment. The FBS contained in the medium for this study was deactivated as described in “2.1 Alkaline Phosphatase (ALP) Activity Assay”.

#### 2.7.1. Preparation of Conditioned Medium from PPU7 and HUCPVCs

The PPU7 or HUCPVCs were separately plated on a culture dish (10 cm ID) at a density of 3.16 × 10^4^ cells/cm^2^. The supernatant, namely mineralization medium after 2 days of culture when cells had reached confluence, was collected by centrifugation and passed through a 0.44 μm filter, which was then used as conditioned medium in the following experiment. 

#### 2.7.2. Preparation of Mineralization Medium Containing Conditioned Medium of PPU7 or HUCPVC

The conditioned medium prepared from PPU7 (CM-PPU7) or HUCPVCs (CM-HUCPVC) was added into a mineralization medium at 20% and 50%.

#### 2.7.3. Comparison of Mineralized Nodules in PPU7 Cultured in Mineralization Medium Containing CM-PPU7 or CM-HUCPVC

PPU7 was grown on a 12-well plate at an initial density of 3.16 × 10^4^ cells/cm^2^. After incubation for 24 h, the mineralization medium was changed to the above mineralization medium containing 20% and 50% CM-PPU7 or CM-HUCPVC. The cells were cultured for up to 7 days. The mineralization was visualized by Alizarin Red S staining using the same method described above (see Section 2.5. Detection of mineralized nodules in HUCPVCs).

For the measurement of Ca concentration, each well on the plates was rinsed with PBS, and the calcium was dissolved in 0.5 mL of 0.5 N HCl with gentle rocking for 1 h. The calcium concentration in the eluate was determined using a calcium assay kit (Calcium E-test Wako, Wako Pure Chemical Industries, Ltd.) and by spectrophotometrically following the color development at 595 nm. All values were normalized against the cultivation area.

### 2.8. Statistical Analysis

For the ALP and MTS assays and the qPCR and calcium analyses, all values are presented as the mean ± standard error of the mean (SEM). Statistical significance was determined using the nonparametric Steel’s test for the ALP and MTS assays, the Steel–Dwass test for qPCR, and the Mann–Whitney U test for calcium analysis. In all cases, *p* < 0.05 was regarded as indicating a statistically significant difference. 

## 3. Results

### 3.1. Examination of Conditions for Producing ALP-Positive HUCPVCs 

**Figure 1:**
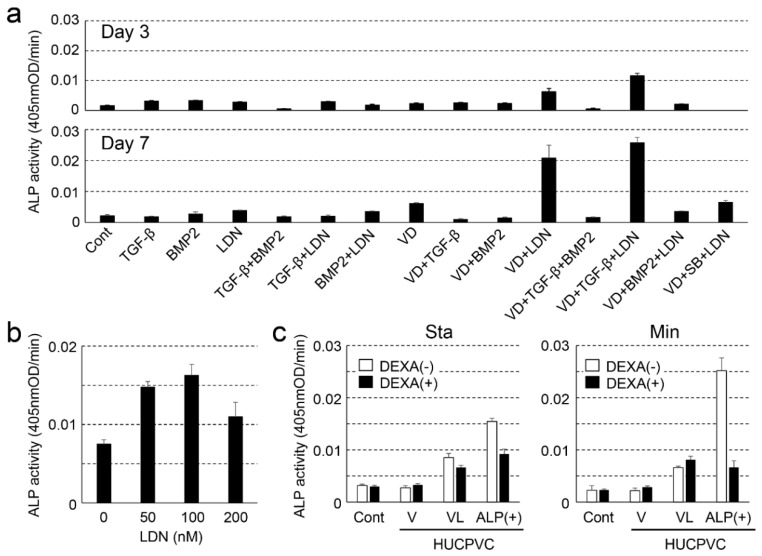
Examination of the optimal conditions for creating alkaline phosphatase-positive HUCPVCs (ALP(+)-HUCPVCs) from HUCPVCs. (**a**) Effect of various factors on the ALP-inducing activity of HUCPVCs. HUCPVCs were cultured with or without various factors, and ALP-inducing activity was measured at days 3 (**top**) and 7 (**bottom**). TGF-β: transforming growth factor-beta1, BMP2: human bone morphogenic protein-2, LDN: LDN-193189, VD: activated vitamin D3, SB: SB431542. Cont: HUCPVCs cultured without factors. (**b**) Effect of LDN-193189 (0, 50, 100, and 200 nM) on ALP-inducing activity of HUCPVCs. (**c**) Effect of dexamethasone (DEXA) on the ALP-inducing activity of HUCPVCs. HUCPVCs were cultured with or without DEXA in the absence of VD, LDN, and TGF-β1 (Cont) or in the presence of activated vitamin D3 alone (V-HUCPVCs); VD and LDN (VL-HUCPVCs); and VD, LDN, and TGF-β1 (ALP(+)-HUCPVCs). Sta: standard medium, Min: mineralization medium. Values are the mean ± standard error of the mean (SEM) of 6 wells.

We first attempted to determine the optimum concentration for the differentiation of HUCPVCs into high alkaline phosphatase activity containing HUCPVCs (ALP(+)-HUCPVCs) because the intrinsically high ALP activity in cells is thought to affect cell growth [24]. To achieve this purpose, we investigated ALP activity under various combinations of TGF-β1, BMP-2, VD, LDN, and SB (Figure 1a). Compared to the control cultured in the absence of VD, LDN, and TGF-β1, the ALP activity showed a tendency of increasing for the combination of VD and LDN or VD, LDN, and TGF-β1 on day 3. In particular, the combination of VD, LDN, and TGF-β1 dramatically enhanced ALP activity on day 7. Based on this result, we decided to focus on HUCPVCs cultured in the presence of VD only (V-HUCPVCs); VD and LDN (VL-HUCPVCs); and VD, LDN, and TGF-β1 (ALP(+)-HUCPVCs) and found that LDN was a key factor for the differentiation of HUCPVCs to ALP(+)-HUCPVCs. We next investigated the effects of different concentrations of LDN on ALP activity in HUCPVCs. The ALP activity at 50 and 100 nM LDN was approximately 2.0-fold higher than that at 0 nM LDN, but its activity level was reduced in 200 nM LDN (Figure 1b). We further attempted to investigate the effect of DEXA because it is known to induce the differentiation of undifferentiated mesenchymal stem cells into osteoblasts and promote the expression of ALP [27]. Interestingly, the ALP activity levels of V-HUCPVCs, VL-HUCPVCs, and ALP(+)-HUCPVCs cultured in either standard or mineralization medium were reduced in the presence of DEXA. Notably, the ALP level of ALP(+)-HUCPVCs was 1.67-fold lower for the standard medium and 3.79-fold lower for the mineralization medium than when DEXA was absent (Figure 1c).

### 3.2. ALP Gene Expression and ALP Staining of HUCPVCs

**Figure 2:**
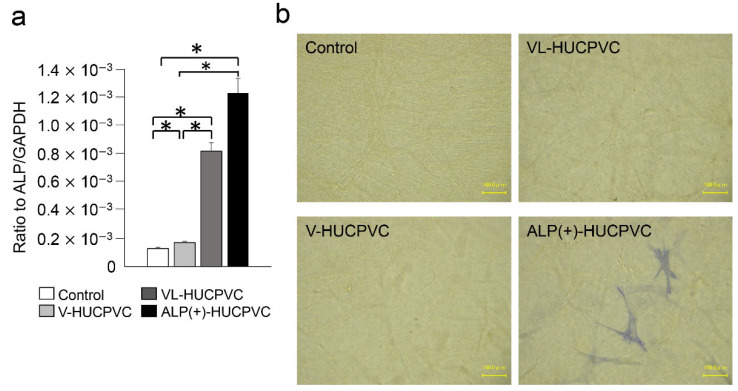
Expression of ALP gene and detection of ALP activity in HUCPVCs. (**a**) qPCR analysis of the *ALP* gene of V-HUCPVCs, VL-HUCPVCs, and ALP(+)-HUCPVC on day 7. The mRNA expression value was normalized to that of the reference gene glyceraldehyde-3-phosphate dehydrogenase (*GAPDH*), and the relative quantification data for *ALP* in HUCPVCs were generated on the basis of a mathematical model for relative quantification in a qPCR system (*n* = 6). All values are presented as the mean ± SEM (* *p* < 0.05, Steel–Dwass test). (**b**) ALP staining of 3D-cultured V-HUCPVCs, VL-HUCPVCs, and ALP(+)-HUCPVCs. HUCPVCs in collagen gel solution were spread on a collagen-coated plate and gelled, followed by culture for 21 days without (Control) or with VD, LDN, and TGF-β1 (Scale bar = 100 μm).

**Figure 3:**
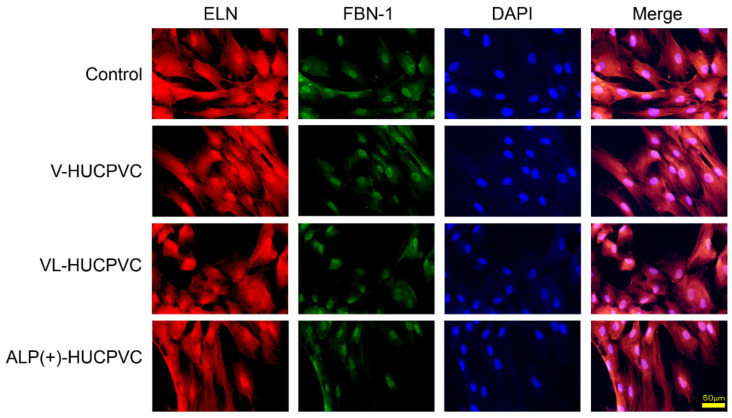
Morphological observation of HUCPVCs by fluorescent immunostaining using elastin and fibrillin-1 antibodies. Fluorescence detection using Alexa Fluor 546-labeled elastin (ELN) and Alexa Fluor 488-labeled fibrillin-1 (FBN-1) into V-HUCPVCs, VL-HUCPVCs, and ALP(+)-HUCPVCs. Each image was compared to HUCPVCs cultured in the absence of VD, LDN, and TGF-β1 (Control). Cells with co-existing elastin and fibrillin-1 were detected in the merged image, which displayed light-red fluorescence (Merge). Elastin (red) and fibrillin-1 (green) in fixed HUCPVCs were detected using human elastin and fibrillin-1 antibodies at a dilution of 1:200, respectively. For secondary antibody application, Alexa Fluor 546-conjugated rabbit anti-mouse IgG (H+L) antibody for elastin and an Alexa Fluor 488 goat anti-rabbit IgG (H+L) antibody for fibrillin-1 were used at 1:500. DAPI was used to stain the cell nuclei (blue) at a concentration of 2 μg/mL (scale bar, 50 μm).

We also investigated the *ALP* expression level and ALP staining of V-HUCPVCs, VL-HUCPVCs, and ALP(+)-HUCPVCs at both the genetic and histomorphological levels. Compared to the control, the level of *ALP* mRNA was significantly increased in both VL-HUCPVCs (6.31-fold) and ALP(+)-HUCPVCs (9.46-fold) (Figure 2a). ALP(+)-HUCPVCs appeared blue-colored in ALP staining images (Figure 2b). Using elastin and fibrillin-1 antibodies, we immunohistologically investigated the morphology of V-HUCPVCs, VL-HUCPVCs, and ALP(+)-HUCPVCs (Figure 3). At 7 days, cells were collected and stained with 4′,6-diamidino-2-phenylindoledihy-drochloride (DAPI), and AlexaFluor 546-labeled anti-elastin and AlexaFluor 488-labeled anti-fibrillin-1 antibodies. When combined with DAPI staining of the nuclei of all fixed HUCPVCs, we found that HUCPVCs possess a fibroblast-like morphology. No significant changes in the HUCPVC morphology were observed in the merged images.

### 3.3. Cell Proliferation Rate of HUCPVCs

**Figure 4:**
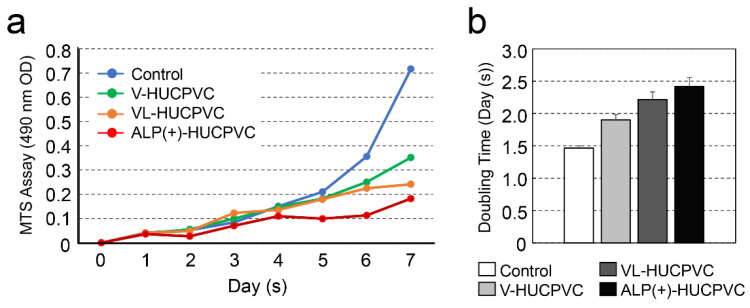
Change in cell proliferation of HUCPVCs. (**a**) Changes in cell proliferation over time by MTS assay. V-HUCPVCs, VL-HUCPVCs, and ALP(+)-HUCPVCs were cultured at a final volume of 120 μL/well for 1 h at 37 °C. MTS reagent was added, and absorbance of 490 nm was recorded using a microplate reader. (**b**) Cell population doubling level against days. Control: HUCPVCs cultured in the absence of VD, LDN, and TGF-β1.

We next investigated the effects on the HUCPVC proliferation rate s (Figure 4a). The proliferation of HUCPVCs cultured under each condition differed after the fourth day. The cell proliferation rate of HUCPVCs was the highest in controls cultured in the absence of VD, LDN, and TGF-β1. Compared to the control on day 7, the cell proliferation rate was reduced approximately 2.04-, 2.97-, and 3.94-fold for V-HUCPVCs, VL-HUCPVCs, and ALP(+)-HUCPVCs, respectively. Surprisingly, the proliferation of ALP(+)-HUCPVCs was the slowest of all conditions. In comparison to the cell doubling level of the control for 7 days, approximately 1.3-, 1.47-, and 1.63-fold days were required for doubling of V-HUCPVCs, VL-HUCPVCs, and ALP(+)-HUCPVCs, respectively (Figure 4b).

### 3.4. Quantitative PCR Analysis of HUCPVCs

**Figure 5:**
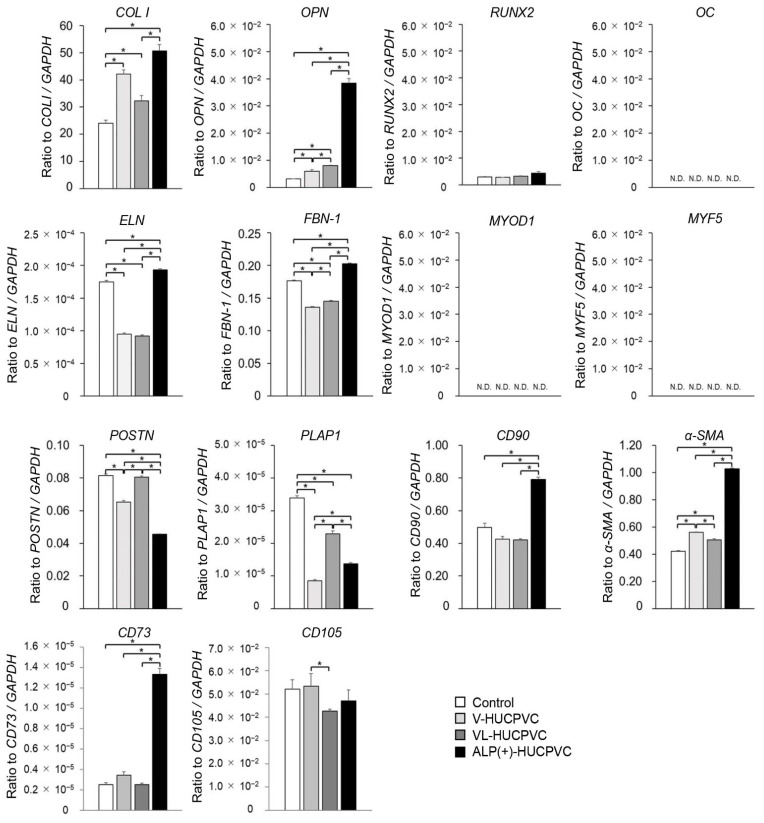
Quantitative PCR (qPCR) analysis of tissue-specific differentiation marker genes for V-HUCPVCs, VL-HUCPVCs, and ALP(+)-HUCPVCs on day 7 after the culture. Control: HUCPVCs cultured in the absence of VD, LDN, and TGF-β1. *COL I*, collagen type I alpha 1 chain; *OPN*, osteopontin; *RUNX2*, runt-related transcription factor 2; *OC*, osteocalcin; *ELN*, elastin; *FBN-1*, fibrillin-1; *MYOD1*, myoblast determination protein 1; *MYF5*, myogenic factor 5; *POSTN*, periostin; *PLAP1*, periodontal ligament associated protein-1; *CD73*, *CD90*, *CD105*, cluster of differentiation 73, 90, 105; *α-SMA*, alpha-smooth muscle actin. ND, not determined. Each mRNA expression value was normalized to that of the reference gene *GAPDH*, and the relative quantification data for *COL I*, *OPN*, *RUNX2*, *OC*, *ELN*, *FBN-1*, *MYOD1*, *MYF5*, *POSTN*, *PLAP1*, *CD90*, *α-SMA*, *CD73*, and *CD105* in HUCPVC were generated on the basis of a mathematical model for relative quantification in a qPCR system (*n* = 6). All values are presented as the mean ± SEM (* *p* < 0.05, Steel–Dwass test).

In addition to the *ALP* gene expression results, we investigated the direction of HUCPVC differentiation at the genetic level. The mRNA levels were analyzed in HUCPVCs on day 7 using qPCR (Figure 5). We quantified the mRNA expression of *COL I*, *OPN*, *RUNX2,* and *OC* as markers for differentiation into bone tissue; *ELN* and *FBN-1* as markers for differentiation into elastic tissue; *MYOD1* and *MYF5* as markers for differentiation into muscle tissue; and *POSTN* and *PLAP-1* as markers for differentiation into periodontal ligament cells. We also analyzed the gene expression of *CD90* and *α-SMA* as myofibroblast markers. Compared with the control, the mRNA levels of *OPN* and *COL I* were significantly higher in V-HUCPVCs (1.86-fold for *OPN* and 1.75-fold for *COL I*) and VL-HUCPVCs (2.48-fold for OPN and 1.34-fold for *COL I*). These mRNA levels of these three genes were considerably higher in ALP(+)-HUCPVCs (11.8-fold for *OPN* and 2.10-fold for *COL I*). The mRNA level of *RUNX2* was not significantly different among V-, VL-, and ALP(+)-HUCPVCs. In addition to the above markers of bone tissue differentiation, the mRNA levels of *ELN* and *FBN-1* were lower in V-HUCPVCs (1.84-fold for *ELN* and 1.90-fold for *FBN-1*) and VL-HUCPVCs (1.90-fold for *ELN* and 1.22-fold for *FBN-1*), but the expression levels were higher in ALP(+)-HUCPVCs (1.11-fold for *ELN* and 1.15-fold for *FBN-1*). Similarly, the mRNA levels of *CD73*, *CD90*, and *α-SMA* were also significantly higher in ALP(+)-HUCPVCs (5.24-fold for *CD73*, 1.59-fold for *CD90*, and 2.43-fold for *α-SMA*). No difference was observed in the expression of *CD105* between ALP(+)-HUCPVC and the control. The mRNA levels of *POSTN* and *PLAP1* were lower in V-HUCPVCs (1.84-fold for *POSTN* and 3.95-fold for *PLAP1*) and VL-HUCPVCs (1.90-fold for *POSTN* and 1.49-fold for *PLAP1*). In particular, these gene expression levels were remarkably lower in ALP(+)-HUCPVCs. No expression of *ON*, *MYOD1*, or *MYF5* was detected. 

### 3.5. Observation of Actin Filaments of HUCPVCs

**Figure 6:**
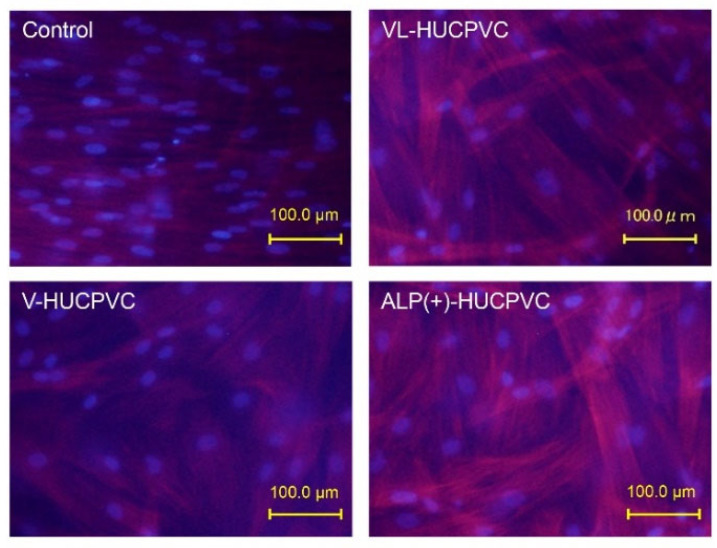
Rhodamine-phalloidin staining of HUCPVCs showing actin cytoskeletal morphology. HUCPVCs in collagen gel solution were cultured in the absence (Control) or presence of VD alone (V-HUCPVCs); VD and LDN (VL-HUCPVCs); and VD, LDN, and TGF-β1 (ALP(+)-HUCPVCs) for 21 days. DAPI was used to stain the cell nuclei (blue) as the counterstaining. Actin filament was stained magenta by rhodamine phalloidin; its staining increased in VL-HUCPVCs and ALP(+)-HUCPVCs (scale bar, 100 μm).

The qPCR results puzzled us in terms of the direction of HUCPVC differentiation, so we examined the actin cytoskeleton of HUCPVCs and conducted an immunohistological experiment using OPN and α-SMA antibodies. It can be seen in Figure 6 that both the control and V-HUCPVCs showed low rhodamine-phalloidin staining of actin. By contrast, both VL-HUCPVCs and ALP(+)-HUCPVCs showed increased magenta staining of actin filaments in spindle-shaped cells. In addition, we were barely able to observe actin filaments in the square-shaped HUCPVCs. 

**Figure 7:**
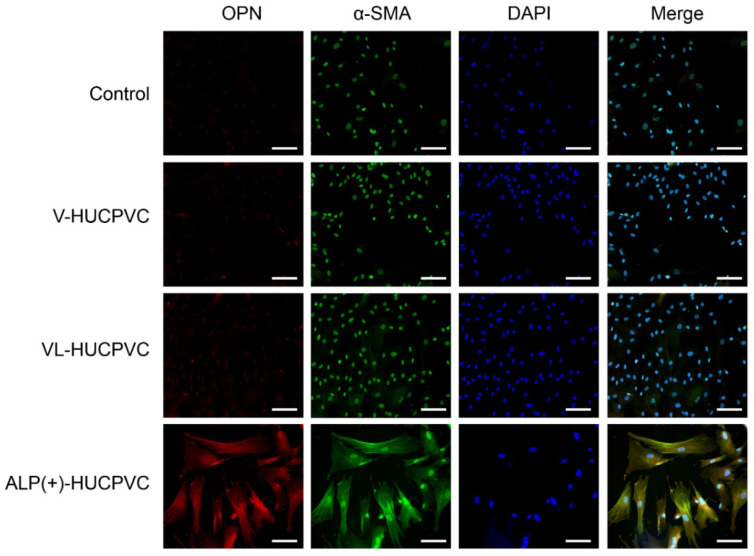
Morphological observation of HUCPVCs by fluorescent immunostaining using osteopontin and α-SMA antibodies. Fluorescence detection of Alexa Fluor 546-labeled osteopontin (OPN) and Alexa Fluor 488-labeled α-SMA (α-SMA) onto V-HUCPVCs, VL-HUCPVCs, and ALP(+)-HUCPVCs. Each image was compared with HUCPVCs cultured in the absence of VD, LDN, and TGF-β1 (Control). Cells with detection of co-existing osteopontin and α-SMA are shown in the merged image, in which overlap is displayed as yellow fluorescence (Merge). Detection of osteopontin (red) and α-SMA (green) in fixed HUCPVCs was carried out using human osteopontin and α-SMA antibodies at a dilution of 1:300. For secondary antibody application, Alexa Fluor 546-conjugated rabbit anti-mouse IgG (H+L) antibody for osteopontin and Alexa Fluor 488 goat anti-rabbit IgG (H+L) antibody for α-SMA were used at 1:500. DAPI was used to stain the cell nuclei (blue) at a concentration of 2 μg/mL (scale bar, 100 μm).

We immunohistologically investigated the morphology of V-HUCPVCs, VL-HUCPVCs, and ALP(+)-HUCPVCs using osteopontin and α-SMA antibodies (Figure 7). Cells were collected and stained with AlexaFluor 546-labeled anti-osteopontin and AlexaFluor 488-labeled anti-α-SMA antibodies with DAPI. When these images were combined with those of DAPI staining of the nuclei of all fixed HUCPVCs, the control, V-HUCPVCs, and VL-HUCPVCs showed little staining for the two antibodies. By contrast, ALP(+)-HUCPVCs clearly displayed staining by both osteopontin and α-SMA antibodies.

### 3.6. Mineralization-Induction Ability of HUCPVCs

**Figure 8:**
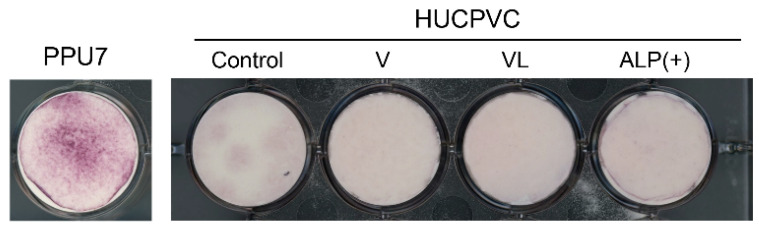
Mineralization-induction ability of HUCPVCs. Alizarin Red S staining for mineralized nodules on day 21. HUCPVCs cultured in the absence (Control) or presence of VD alone (V-HUCPVC); VD and LDN (VL-HUCPVC); and VD, LDN, and TGF-β1 (ALP(+)-HUCPVC). A porcine dental-pulp-derived cell line, PPU7, was also cultured in mineralization medium for 7 days and used as the positive control (PPU7). No mineralized nodules formed in the HUCPVC group but are clearly displayed in PPU7.

**Figure A1:**
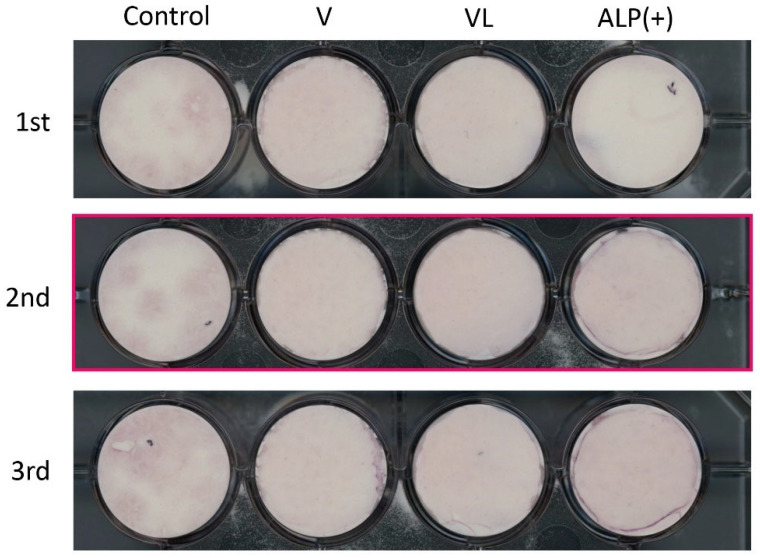
Mineralization induction ability of HUCPVC. The experiment was repeated 3 times (1st, 2nd and 3rd). The cropped part that is shown in the main Figure 8 is marked by a red box.

**Figure A2:**
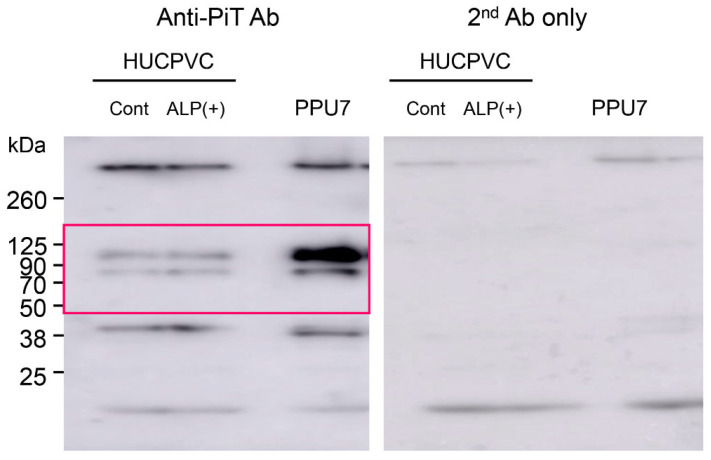
Uncropped image of all Western blots shown in the main Figure 9c. The cropped part that is shown in the main Figure is marked by a red box.

Since the mRNA levels of *ALP*, *OPN*, and *COL I*, as the differentiation markers of osteoblasts, were enhanced in ALP(+)-HUCPVCs, we investigated whether HUCPVCs possessed mineralization-induction ability. We evaluated the nodule formation and mineralization-induction ability of cells by Alizarin Red S staining after the culture of HUCPVCs in mineralization-inducing medium (Figure 8). We also used PPU7 as a positive control; the plate of PPU7 cultured in mineralization medium for 7 days (i.e., positive control) displayed mineralized nodules with Alizarin Red S staining. By contrast, at 21 days after mineralization induction, no mineralized nodules were observed in HUCPVCs under any of the conditions (Figure A1).

### 3.7. Characterization of Matrix Vesicle in HUCPVCs

**Figure 9:**
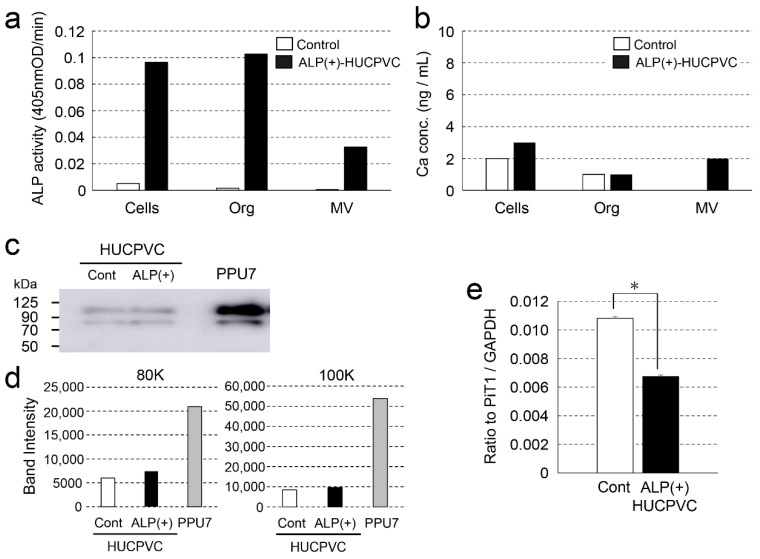
Characterization of matrix vesicles of ALP(+)-HUCPVCs. ALP(+)-HUCPVCs were separated into three fractions, whole cells, organelles, and matrix vesicles, by ultracentrifugation. HUCPVCs cultured in the absence of VD, LDN, and TGF-β1 were also fractionated and used as the control (Control). (**a**,**b**) ALP-inducing activity and calcium contents in the three fractions Cells: whole cells, Org: organelles, and MV: matrix vesicles. (**c**) Western blot using the PiT1 antibody. Two PiT1-positive bands having a molecular weight of approximately 80 and 100 kDa were observed in the MVs of HUCPVCs. Matrix vesicles of PPU7 (PPU7) were also prepared and used for comparison with HUCPVCs. (**d**) Quantification analysis of two PiT1-positive bands on Western blot analysis. The area of each PiT1-positive band (80 and 100 kDa) was determined using ImageJ densitometry software. (**e**) qPCR analysis of the *PiT1* gene of HUCPVCs. HUCPVCs were cultured in the absence (Cont) or the presence (ALP(+)-HUCPVCs) of VD, LDN, and TGF-β1 for 7 days, and mRNA was prepared and analyzed by qPCR. The mRNA expression value was normalized to that of the reference gene glyceraldehyde-3-phosphate dehydrogenase (*GAPDH*), and the relative quantification data for *PIT1* in HUCPVCs were generated on the basis of a mathematical model for relative quantification in a qPCR system (*n* = 6). All values are presented as the mean ± SEM (* *p* < 0.05, Steel–Dwass test).

To obtain information regarding why nodule formation did not occur, we attempted to characterize the matrix vesicles (MVs) in HUCPVCs. The ALP activity in three fractions (whole cells, organelles, and MVs) of ALP(+)-HUCPVCs was higher than that in the control (18.7-fold for cells, 61.6-fold for organelles, and 49.0-fold for MVs) (Figure 9a). The Ca concentration in two fractions, whole cells and MVs, was also higher in ALP(+)-HUCPVCs (Figure 9b). Next, we focused on the MV fraction and analyzed inorganic phosphate transporter 1 (PiT1) at both the protein and genetic levels. We performed a Western blot analysis to detect PiT1 in MVs prepared from ALP(+)-HUCPVCs. We also prepared MVs from PPU7 to compare the protein levels of PiT1. Two bands of PiT1 at approximately 80 and 100 kDa were detected in the MVs of both PPU7 and HUCPVCs in the Western blot analysis (Figure 9c). In the HUCPVC group, the intensities of the two bands of 80 and 100 kDa were almost the same in both the control and ALP(+)-HUCPVCs. Compared to PPU7, the intensity of PiT1 of PPU7 was remarkably higher than that of ALP(+)-HUCPVCs (2.82-fold for 80 kDa and 5.62-fold for 100 kDa) (Figure 9d). In the genetic-level experiments, the mRNA level of *PiT1* in ALP(+)-HUCPVCs was significantly lower compared to that in the control (1.60-fold) (Figure 9e). 

### 3.8. Effect of CM-PPU7 and CM-HUCPVC on Mineralization-Induction Ability

**Figure 10:**
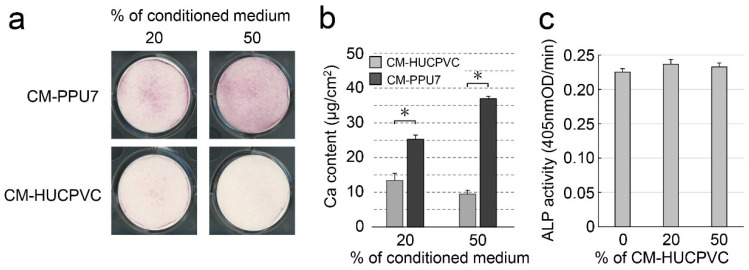
Possibility of the presence of anti-mineralization substances produced by HUCPVCs. PPU7 cells were cultured in mineralization medium containing 20% or 50% conditioned medium from PPU7 (CM-PPU7) or from HUCPVCs (CM-HUCPVC). (**a**) Alizarin Red S staining for nodule precipitates on day 7. PPU7 cultured with CM-PPU7 displayed mineralized nodules but showed inhibited nodule formation when cultured with CM-HUCPVC. (**b**) Calcium contents in PPU7 were determined on day 7 after mineralization induction. The amount of calcium was higher in PPU7 cultured with CM-PPU7, but lower in that cultured with CM-HUCPVC. Values are the means ± SEM of 6 culture wells. Asterisk (*) indicates a significant difference (* *p* < 0.05, Mann–Whitney U test) between CM-HUCPVC and CM-PPU7. (**c**) ALP-inducing activity of PPU7 cultured with CM-HUCPVC. PPU7 exhibited the same level of ALP activity regardless of the CM-HUCPVC concentration.

To further understand the ability of HUCPVCs to suppress mineral induction, we compared the mineralization-induction ability of PPU7 cells cultured in mineralization medium containing CM-PPU7 and CM-HUCPVC (Figure 10a). When PPU7 cells were cultured in mineralization medium containing CM-PPU7, the plate of cells stained by Alizarin Red S clearly displayed precipitated nodules by depending on the concentration of CM-PPU7. By contrast, when PPU7 cells were cultured in mineralization medium containing CM-HUCPVC, nodule formation was dramatically inhibited. The amount of Ca in PPU7 cultured in CM-PPU7 was significantly higher than that in CM-HUCPVC (1.89-fold for 20% CM and 3.91-fold for 50% CM; Figure 10b). The ALP activity of PPU7 cells cultured in mineralization medium containing CM-HUCPVC appeared to be at the same level regardless of the CM-HUCPVC concentration (Figure 10c).

## 4. Discussion

The different types of pluripotent stem cells, such as mouse and human embryonic stem cells, embryonic germ cells, and embryonic carcinoma cells, each require different growth environments for self-replication and express different cell surface markers. ALP is widely used as a common stem cell marker of the aforementioned stem cell types [23]. Our previous study showed that intrinsically high ALP activity promoted the differentiation of dental pulp cells into odontoblast-like cells [24]. Thus, we conceived that ALP may be a common key enzyme in the different growth environments of cells. Our initial purpose in this study was to create HUCPVCs with high ALP activity and to determine the optimum conditions that increase ALP activity in HUCPVCs. In addition to ALP, cytokines such as BMP and TGF-β are thought to be involved in the regulation of the differentiation of cells such as hard tissue-forming cells [28,29,30,31,32,33]. We revealed that the combination of VD and LDN increased the ALP activity and *ALP* mRNA levels in HUCPVCs (VL-HUCPVCs), which were further enhanced by the addition of TGF-β1 in ALP(+)-HUCPVCs. We morphologically demonstrated that ALP(+)-HUCPVCs possess ALP activity in cells. These findings suggest that the culture of HUCPVCs suppresses BMP activity, and that adding TGF-β1 leads to the induction of differentiation into ALP(+)-HUCPVCs. 

Fibroblasts are one of the typical cells that compose connective tissue and are characterized by flat or spindle-shaped morphology with a large number of cytoplasmic protrusions. Fibroblasts mainly produce type I collagen, elastin, and hyaluronic acid to build the dermis. We observed that the morphology of HUCPVCs is basically fibroblast-like and the morphology ALP(+)-HUCPVCs induced in the presence of VD, LDN, and TGF-β1 was similar to that of others induced under alternative conditions. However, we found that ALP(+)-HUCPVCs possess high mRNA levels of *COL I*, *ELN*, *FBN-1*, *CD73*, and *CD90*. These findings suggest that ALP(+)-HUCPVCs are morphologically similar to HUCPVCs, but somewhat different in nature. 

In the process of skeletal muscle development, MSCs differentiate into myoblasts, and myoblasts fuse with each other to differentiate into multinucleated myotubes, followed by contractile myofibers. Genes called muscle regulatory factors, such as *MYOD1* and *MYF5*, play an important role in myoblast differentiation [34]. We observed minimal detection of *MYOD1* and *MYF5* expression in HUCPVCs. This result suggests that HUCPVCs possess a limited abilities to differentiate into skeletal muscle cells.

Regarding periodontal tissue, fibroblasts containing high ALP activity in the gingiva of adult periodontitis patients show slow cell proliferation but retain collagen productivity [35]. Periodontal ligament cells containing both high and low ALP activities (ALP(+)PDL and ALP(−)PDL, respectively) coexist in periodontal ligament tissue. Of these, the cell proliferation of ALP(+)PDL is slower than that of ALP(−)PDL, but ALP(+)PDL appears as an osteoblast-like cell population [36]. We demonstrated that the proliferation of ALP(+)-HUCPVCs was significantly slower, but the mRNA levels of *COL I* and *OPN* were elevated, although *RUNX2* gene expression showed no significant change. Our findings suggest that ALP(+)-HUCPVCs may have both fibroblast- and osteoblast-like characteristics. As the gene expression of *OC* was barely detectable, the differentiation of osteoblasts might occur in the early stages. In addition, we revealed that the combination of VD, LDN, and TGF-β1 suppressed the gene expression of *POSTN* and *PLAP1*, which are markers of differentiation into periodontal ligament cells. This result suggests that ALP(+)-HUCPVCs do not appear to differentiate into periodontal ligament-like cells. 

The initiation of the differentiation of myofibroblasts from fibroblasts is mainly regulated by TGF-β1, followed by the induction of α-SMA and type I collagen [37,38,39,40]. α-SMA belongs to the actin family and is mainly present in smooth muscle cells. In organ fibrosis, activated fibroblasts—i.e., myofibroblasts—accumulate at the fibrosis site and express high levels of α-SMA with the production of type I collagen and osteopontin. In addition to markers of myofibroblast formation, such as α-SMA, CD73 and CD90 also regulates proliferation and differentiation in fibroblasts, so activated fibroblasts, such as myofibroblasts, express *CD73* and *CD90* [41]. Morphologically, myofibroblasts possess more actin filaments, forming robust actin stress fibers in the protoplasm [42]. Fibroblasts secrete various extracellular matrices; in particular, osteopontin promotes TGF-β1 activation and myofibroblast differentiation [43]. In pulmonary fibrosis, osteopontin-positive fibroblasts accumulate at the beginning of fibrosis [44]. We demonstrated that ALP(+)-HUCPVCs showed significantly higher mRNA levels of *COL I*, *OPN*, *CD73*, and *CD90*. In addition to the above genetic study, our histological and immunohistochemical studies revealed that the increases in actin filaments and stress fibers, consisting of osteopontin and α-SMA, were clearly present in ALP(+)-HUCPVCs. Taken together, our results suggest that the inclusion of TGF-β1 in culture causes the transition of HUCPVCs toward adopting a myofibroblast phenotype, such as in the case of ALP(+)-HUCPVCs. 

In in vitro studies, hard tissue-forming cells such as C2C12, MC3T3-E1, and PPU7 have generally been observed to form mineralized nodules when they are cultured in a mineralization medium [24,45,46]. When HUCPVCs were cultured in conditioned media derived from cultures of human-bone-marrow derived mesenchymal stromal cells, mineralized nodules, suggestive of osteogenic differentiation, formed in HUCPVCs [19]. Following a 21-day culture, we observed that no mineralized nodules formed in any HUCPVCs cultured with or without VD, LDN, and TGF-β1. This finding strongly suggests that the mineralization medium normally used for hard tissue-forming cells does not induce the mineralization of HUCPVCs.

The mechanism of mineralized and non-mineralized cells has not yet been elucidated. For mineralized cells, MVs germinated from hard tissue-forming cells, such as osteoblasts and odontoblasts, play an important role in the mechanism of mineralization initiation of mesenchymal hard tissue. Tissue-nonspecific ALP (TNAP) is an enzyme localized on the cell surface of MVs as a membrane-anchored enzyme and hydrolyzes pyrophosphate into phosphate ion monomers. Subsequently, monophosphate ions (PO_4_^3−^) are transported into MVs through PiT1 located on the membrane of MVs, and the nucleation of calcium phosphate crystals is induced by PO_4_^3−^ and Ca^2+^ accumulated inside the MVs [47,48]. To investigate PiT1 at both the genetic and protein levels, we first prepared the MV fraction of HUCPVCs because TNAP can be found in cellular components other than MVs. Using the MV fraction obtained by ultracentrifugation, we found that ALP(+)-HUCPVCs possess TNAP activity in MVs and the amount of PiT1 in the MVs of ALP(+)-HUCPVCs was dramatically lower compared to in the MVs of PPU7. In addition, we revealed that the gene expression of PiT1 in ALP(+)-HUCPVCs was suppressed compared to in PPU7. These findings suggest that the nucleation of phosphate calcium crystals does not proceed because of the restricted uptake of PO_4_^3−^, generated by TNAP, into the MVs. 

Despite the presence of osteoblast-like cells in periodontal ligament tissue, this tissue maintains tissue homeostasis without mineralization. Components of the extracellular matrix, such as biglycan and decorin, are listed as candidates for this regulator; they may regulate osteoblast-like differentiation and mineralization in periodontal ligament [49,50,51]. In particular, it was shown that periodontal ligament cells containing high ALP activity (ALP(+)PDL) present in the periodontal ligament are promoted and regulated into osteoblast-like cells by biglycan [36]. We demonstrated that the culture media containing CM-PPU7 promoted the formation of mineralized nodules of PPU7, but that with CM-HUCPVC dramatically suppressed mineral induction. This finding indicates that in CM-HUCPVC, a substance that acts an inhibitor of mineral induction in PPU7 is present. Moreover, it is possible that HUCPVCs did not show the ability to induce mineralization due to this inhibitor. In addition, as our ALP(+)-HUCPVCs did not show mineralization-induction ability, some substance other than biglycan and decorin, associated with the above ALP(+)PDL, may be involved in the suppression of mineralization. Identification of this substance may help elucidate the causes not only of hard tissue-forming diseases in the oral region, but also ectopic calcification throughout the body.

In general, many fibroblasts randomly exist in the stroma, whereas myofibroblasts are normally present with cell morphology specific to organs such as the liver, lung, skin, kidney, and those of the gastrointestinal system. Myofibroblasts morphologically transform into activated myofibroblasts by increasing the expression of α-SMA, extracellular matrix proteins, and cytokines to impair stress. Organ fibrosis involves scarring and tissue hardening caused by the excessive deposition of extracellular matrix proteins produced by myofibroblasts in response to chronic inflammation [52,53,54].

In the future, the ALP(+)-HUCPVCs we created from HUCPVCs in the present study may be used as a model for myofibroblast research, contributing not only to studying rapid connective tissue healing but also to elucidating their potential uses, such as for organ fibrosis and drug development. Furthermore, we think that these ALP(+)-HUCPVCs are a potential source of ligament and tendon tissue for patients with ligament injuries, and hope that they can contribute to the application of therapies in the orthopedic field. In terms of their application in dental treatment, ALP(+)-HUCPVCs may contribute to the treatment of gingival recession. As periodontal disease progresses, gingival recession also progresses, but after treatments such as free gingival flap transplantation and curettage, the regressed gingival margin gradually returns to its original position. This restorative change for the regressed gingiva, called creeping attachment, requires the presence of myofibroblasts in the gingival tissue. Gingival recession is also caused by incorrect tooth brushing, orthodontic treatment, and aging, whereby the exposed root surface is susceptible to tooth caries. Thus, it is possible that the ALP(+)-HUCPVCs we created, displaying the features of myofibroblasts, may be useful for the treatment of gingival recession such as through gingivoplasty. Further studies and animal experiments using HUCPVCs are required to assess these possibilities.

## 5. Conclusions

In the present study, we successfully produced myofibroblast-like ALP(+)-HUCPVCs from HUCPVCs via TGF-β1 stimulation. Moreover, we found evidence that HUCPVCs may secrete an inhibitor of hard tissue formation. Thus, our findings show that HUCPVCs may serve as a model in studies exploring rapid connective tissue healing, tissue fibrosis treatment, and the causes of ectopic mineralization. 

## Data Availability

Not applicable.

## Abbreviations

MHC-II	major histocompatibility complex class II molecules
LDN	4-[6-[4-(1-piperazinyl)phenyl]pyrazol [1,5-a]pyrimidin-3-yl]-quinolinehydrochloride
SB	4-[4-(1,3-benzodioxol-5-yl)-5-(2-pyridinyl)-1H-imidazol-2-yl]-benzamide
MTS	3-(4,5-dimethylthiazol-2-yl)-5-(3-carboxymethoxyphentl)-2-(4-sulfophenyl)-2H-tetrazolium, inner salt)
SDS	sodium dodecyl sulfate
PAGE	polyacrylamide gel electrophoresis
PVDF	polyvinylidene difluoride
ID	internal diameter

## Appendix A

### Preparation of HUCPVC

HUCPVCs were harvested from umbilical cords obtained from consenting patients who had undergone full-term cesarean sections based on ethical approval obtained from the University of Toronto and University Health Network (Toronto, ON, Canada). The cords were retrieved immediately after delivery, and cells were harvested by Tissue Regeneration Therapeutics, Inc., (Toronto, ON, Canada) as follows: Human umbilical cords from full-term patients were obtained immediately upon delivery in a vessel containing 80% Eagle minimum essential medium and alpha modification (Gibco, Burlington, ON, Canada) with 20% antibiotics (167 U/mL penicillin G (Sigma-Aldrich, Oakville, ON, Canada), 50 mg/mL gentamicin (Sigma-Aldrich), and 0.3 mg/mL amphotericin B (Sigma-Aldrich)). Sections of human umbilical cords (4–5 cm in length) were dissected by first separating the epithelium of the section along its length to expose the underlying Wharton’s jelly (WJ). Vessels were pulled away from the surrounding WJ matrix and placed into a 50 mL tube containing 1 mg/mL collagenase (Sigma-Aldrich) in phosphate-buffered saline (PBS) at 37 °C. Following 18–24 h, vessels were removed from the suspension, which was then diluted with PBS to reduce the viscosity of the suspension, and centrifuged. The HUCPVCs were transported at the temperature of liquid nitrogen to Tsurumi University, where they were cultured according to the method of Ennis et al. [55], where clusters of differentiation (CD) of 105^+^, 90^+^, 73^+^, 45^−^, and 34^−^ and MHC-II^−^ phenotypes were revealed by flow cytometry.

## Appendix B

**Table A1:** Selected primers, amplified products and conditions for qPCR analysis in Figure 2a, Figure 5 and Figure 9e.

Gene		Sequence (5′---3′)	Product Size (bp)	qPCR Protocol (45 Cycles)		
*ALP*	F	GGACCATTCCCACGTCTTCA	128			
	R	CAGGCCCATTGCCATACA				
*COL1a1*	F	CGAGAGCATGACCGATGGAT	141			
	R	ACGCTGTTCTTGCAGTGGTA				
*OPN*	F	ACACATATGATGGCCGAGGTGA	116			
	R	GTGTGAGGTGATGTCCTCGTCTGTA				
*RUNX2*	F	CTTTGTAGCACAAACATTGCTGGA	122			
	R	CAAAGCTGTGGTACCTGTTCTGGA				
*OC*	F	CAGAGTCCAGCAAAGGTGCAG	178			
	R	ATGTGGTCAGCCAACTCGTC				
*ELN*	F	CTTGGAGGTGTCCTAGGGGGT	80			
	R	ATGGGAGACAATCCGAAGCCA				
*FBN-1*	F	TCAATGGCTACCCCAAACGG	128	Denaturation	95 °C	10 s
	R	GGCTGTCTTCTCAACATCCCA				
*MYOD1*	F	CGACGGCATGATGGACTACA	134	Annealing	60 °C	10 s
	R	GGCAGTCTAGGCTCGACAC				
*MYF5*	F	GGCATGCCCGAATGTAACAG	150	Extension	72 °C	15 s
	R	GGTGATCCGGTCCACTATGT				
*POSTN*	F	ACACACCCGTGAGGAAGTTG	80			
	R	TCACTGAGAACGACCTTCCCT				
*PLAP1*	F	TCCCAAATCATTAGCAGAACTCA	145			
	R	AAATGCCCCTGGCTCTATCC				
*CD90*	F	TCTCCTCCCAGAACGTCACA	137			
	R	GGACATGAAATCCGTGGCCT				
*α-SMA*	F	GGCTCTGGGCTCTGTAAGGC	142			
	R	TCTGTGCTTCGTCACCCACG				
*PIT1*	F	CCTCCATAAGGCAGATCCAGT	147			
	R	AGGATGGTACCCCACAGAGG				
*CD73*	F	GCAACATGGGCAACCTGATT	133			
	R	TTGCGTTCATCAATGGGCGA				
*CD105*	F	AAGGTGCACTGCCTCAACAT	148			
	R	CAGGAACTCGGAGACGGATG				
*GAPDH*	F	CCATCACCATCTTCCAGGAG	346			
	R	ACAGTCTTCTGGGTGGCAGT				

F: forward, and R: reverse.
